# Errors in Diagnosing Infectious Diseases: A Physician Survey

**DOI:** 10.3389/fmed.2021.779454

**Published:** 2021-11-17

**Authors:** Mahboubeh Haddad, Fereshte Sheybani, HamidReza Naderi, Mohammad Saeed Sasan, Mona Najaf Najafi, Malihe Sedighi, Atena Seddigh

**Affiliations:** ^1^Department of Infectious Diseases and Tropical Medicine, Faculty of Medicine, Mashhad University of Medical Sciences, Mashhad, Iran; ^2^Department of Pediatrics, Faculty of Medicine, Mashhad University of Medical Sciences, Mashhad, Iran; ^3^Clinical Research Unit, Faculty of Medicine, Mashhad University of Medical Sciences, Mashhad, Iran; ^4^Faculty of Medicine, Mashhad University of Medical Sciences, Mashhad, Iran

**Keywords:** infectious diseases, diagnostic errors, cognitive biases, patient safety, survey

## Abstract

**Background:** Infectious diseases are commonly missed or misdiagnosed. Errors in diagnosing infectious diseases not only affect the patient but also the community health.

**Objectives:** To describe our investigation on the most common errors in diagnosing infectious diseases and their causes according to the physicians' reports.

**Methods:** Between August 2018 and February 2019, specialist physicians and residents across Mashhad, Iran were invited to participate in a survey to report errors they had made or witnessed regarding the diagnosis of infectious diseases.

**Results:** Overall, 465 cases were reported by 315 participants. The most common infectious diseases affected by diagnostic errors were upper respiratory tract infections (URTIs) (*n* = 69, 14.8%), tuberculosis (TB) (*n* = 66, 14.1%), pleuro-pulmonary infections (*n* = 54, 11.6%), central nervous system (CNS) infections (*n* = 51, 10.9%), and urinary tract infections (*n* = 45, 9.6%). Errors occurred most frequently in generating a diagnostic hypothesis (*n* = 259, 55/7%), followed by history taking (*n* = 200, 43%), and physical examination (*n* = 191, 41/1%). Errors related to the diagnosis of TB (odds ratio [OR]: 2.4, 95% confidence interval [CI]:0.9–5.7; *P* value: 0.047) and intra-abdominal infections (OR: 7.2, 95% CI: 0.9–53.8; *P* value: 0.02) were associated with more-serious outcomes.

**Conclusion:** A substantial proportion of errors in diagnosing infectious diseases moderately or seriously affect patients' outcomes. URTIs, TB, and pleuropulmonary infections were the most frequently reported infectious diseases involved in diagnostic error while errors related to the diagnosis of TB and intraabdominal infections were more frequently associated with poor outcomes. Therefore, contagious and potentially life-threatening infectious diseases should always be considered in the differential diagnosis of patients who present with compatible clinical syndromes.

## Introduction

Medical errors account for significant clinical and economic burdens and are among the greatest challenges for health care systems (1, 2). The World Health Organization (WHO) has recently prioritized the importance of patient safety and the significance of medical errors (3). Medical errors are a major concern for both patients and healthcare systems. In the United States, an estimated 40,000–80,000 annual in-hospital deaths and 19.5 billion dollars for additional medical services result from diagnostic errors (4, 5).

Diagnostic errors are among the most common medical errors and emerge when a diagnosis is missed, inappropriately delayed, or is wrong (6). About half of the adverse events related to diagnostic errors result in a major disability that is significantly higher than the rates of serious disability due to technical complications of surgery and drug-related adverse events (7). Compared to other types of medical errors, diagnostic errors lead to death and disability almost twice as often as other error categories and account for the highest proportion of total payments among malpractice claims (8). Likewise, autopsy-based studies have highlighted the occurrence of high rates of the discrepancy between clinical and autopsy diagnosis in critically ill patients who succumbed to death (9).

The most common conditions affected by diagnostic discrepancies are infectious diseases, malignant neoplasms, cardiovascular disorders, and complications of physical trauma (10–12). Investigations on the incidence of the most common diagnostic errors in each specialty of medicine and trying to understand their causes and recognize where failures in the diagnostic process occur are valuable efforts to reduce errors and improve patients' safety (13). Infectious disease specialists are frequently consulted about diagnostic and therapeutic approaches of more challenging patients (13). Having a basic understanding and knowledge of clinical reasoning and common cognitive biases are important for those who are dedicated to the management of infectious diseases. Here, we describe our investigation on the most common errors in diagnosing infectious diseases and their causes according to the physicians' reports.

## Methods

Between August 2018 and February 2019, a random cohort of 707 specialist physicians and residents across Mashhad, Iran invited to participate in the survey. The specialty areas of the participants included infectious diseases and tropical medicine, pediatric infectious diseases, internal medicine, pediatrics, and emergency medicine. Mashhad is the second largest city of Iran that is located in the northeast of the country, with 3,001,184 population (14). According to the Iranian Medical Council Registry in 2017, 5,000 general practitioners and 2,800 specialist physicians work in Mashhad.

Using a six-item questionnaire that was previously introduced by Schiff et al. (15), we asked participants to report any cases in which they personally committed or observed what they considered to be errors related to the diagnosis of infectious diseases. It either could be missed or delayed diagnosis of an infectious disease or misdiagnosis of an infectious disease. A missed or delayed diagnosis was defined as a diagnosis that was missed or unintentionally delayed when sufficient information was available. A misdiagnosis was defined as an incorrect diagnosis of a disease before the correct one was made (16).

The questionnaire included six questions regarding the correct diagnosis that should have been made, description of error/failure that was occurred, factors contributing to the occurrence of the error, whether the error was committed or observed by the respondent, the seriousness of the clinical impact, the frequency of occurrence of that type of error seen by the respondent, and respondent's specialty area and the institution they work for. To prevent the possible decline in the response rate of our participants, we did not include the question of “who made the error?” in the survey. Furthermore, questionnaires were anonymous and participants' personally-identifying information was not requested or recorded during data gathering and analysis (Table 1).

**Table 1:** Six-item questionnaire.

1. Diagnosis
What was the (correct) diagnosis that should have been made?
2. What went wrong?
Describe the error/failure that occurred.
3. Clinical impact/outcome
How serious was the clinical impact?
No impact (no impact at all)
Minor (patient inconvenience, dissatisfaction)
Moderate (short-term morbidity, increased length of hospital stay, higher level of care, invasive procedure)
Major (death, permanent, disability, or near life-threatening event)
4. How frequent?
How often do you see this type of error made?
Rare (1 or 2 cases seen)
Infrequent (1 case seen every few years)
Occasional (about 2 or more cases seen each year)
Common (about 3 or more cases seen each month)
5. Institution
Public hospital
Private outpatient clinic
Private hospital
Public hospital outpatient clinic
6. Specialty

Since we translated the questionnaire from English to Persian, the content validity of the questions was checked again by testing with 12 physicians. Furthermore, two of the researchers (AS and MS) invited the physicians to enroll in the survey by visiting them in their clinic or the hospital they worked for and presented an envelope containing the following items to each participant: (1) five questionnaires, (2) an invitation letter describing the research objectives, (3) a list of infections and infectious diseases retrieved from the table of content of the textbook of infectious diseases, entitled “Mandell, Douglas, and Bennett's Principles and Practice of Infectious Diseases 2019”. The envelope was designed with the picture of a newspaper containing short statements about the importance and prevalence of medical errors worldwide. The participants were asked regarding any questions or problems they had about our research and the questionnaire to ensure they understand the purpose of the research and they will answer questions without difficulty in understanding the content of the questionnaires. Researchers answered their questions face-to-face for clarifications whenever a question seems unclear to the participants. Furthermore, the participants had access to the researchers for asking any questions regarding the questionnaires or other items they received. After returning the completed questionnaires, the information provided in each questionnaire was extracted. Using the Diagnostic Error Evaluation and Research (DEER) classification, two researchers (FS and MH) categorized the reported cases based on where the error(s) had occurred in the diagnostic process. If there were any doubt regarding what and where diagnostic errors occurred, a third researcher (HN) was asked for his opinion.

We also coded the cases based on whether it was a misdiagnosis or a missed or delayed diagnosis. In our study, a misdiagnosis could be considered if an infectious disease was mistaken for another infectious or non-infectious disease (e.g., necrotizing fasciitis is being mistaken for deep vein thrombosis), or another infectious disease in the same category (e.g., necrotizing fasciitis is mistaken for cellulitis). It could also be an incorrect diagnosis of an infectious disease instead of another infectious or non-infectious disease (e.g., a patient is misdiagnosed with lymphoma instead of EBV mononucleosis), or another infectious disease in the same category (e.g., a patient is misdiagnosed with streptococcal pharyngitis instead of EBV mononucleosis).

The seriousness of clinical impact was scaled as no impact, minor (i.e., patient inconvenience, dissatisfaction), moderate (i.e., short-term morbidity, increased length of hospital stays, higher level of care or invasive procedure), and major impact (i.e., death, permanent disability, or near life-threatening event).

The frequency of occurrence of a specific type of error, based on the experience of the respondent was scaled as rare (i.e., one or two cases seen), infrequent (i.e., one case seen every few years), occasional (i.e., two or more cases seen each year), and common (i.e., three or more cases seen each month).

### Statistics

Categorical data were described with frequency and percentage. Fisher's exact test and Chi-square tests were used for categorical variables, as appropriate. Logistic regression was used to identify the association between errors in each category of infectious diseases and their impact on patients' clinical outcomes, calculating odds ratio (OR) and their 95% confidence interval (CI). A *p*-value < 0.05 was considered statistically significant.

### Ethics

The Ethics Committee of Mashhad University of Medical Sciences approved the present study assigned with the IR.MUMS.fm.REC.1396.413 Code.

## Results

A total of 541 completed questionnaire(s) were received from 315 survey-eligible physicians (response rate: 44.6%), including 117 (37.1%) specialists or residents in pediatrics or pediatric infectious diseases, 109 (34.6%) in internal medicine, 51 (16.2%) in infectious diseases and tropical medicine, and 38 (12%) emergency medicine. Two hundred fourteen (67.9%) participants worked at public hospitals, 94 (29.8%) private outpatient clinics, 23 (7.3%) private hospitals, and 21 (6.6%) in public hospital outpatient clinics.

Seventy-six questionnaires were excluded either because the reported errors were not related to infectious diseases or they provided incomplete or inconclusive information.

Of 465 cases that were analyzed, 200 (43%) cases were reported to be seen occasionally, 153 (33%) infrequently, 79 (17%) rarely, and 33 (7%) commonly. The seriousness of diagnostic errors was reported as moderate in 288 (62%) of 465 cases, major in 98 (21%), and minor in 79 (17%), and no case (0%) with no impact.

Categories of infectious diseases that were reported to be confounded by diagnostic errors are listed in Table 2, based on the order of frequency. In 216 (46.5%) of 465 cases, the diagnosis of an infectious disease was missed, in 15 (3.2%) cases it was delayed, and in 234 (50.3%) it was misdiagnosed (Table 3). The rendered diagnosis of upper respiratory tract infections (URTIs) was incorrect in 40 (57.9%) of 69 URTI-related errors and it was misdiagnosed instead of other infectious or non-infectious diseases, whereas in 23 (33.3%) cases a wrong causative pathogen was considered (e.g., bacterial instead of viral etiology) and in 6 (8.7%) cases it was missed or diagnosed with delay.

**Table 2:** Infectious diseases affected by diagnostic errors according to the physician reports in order of frequency.

	**Category of infectious diseases**	**As a correct diagnosis of the patient**	**As an incorrect diagnosis which was made**	**Total**
1	URTIs and bronchitis	23 (4.9)^*^	46 (9.9)	69 (14.8)
2	Tuberculosis	51 (10.9)	16 (3.4)	67 (14.7)
3	Pleuro-pulmonary infections	29 (6.2)	25 (5.4)	54 (11.6)
4	CNS Infections	39 (8.4)	13 (2.8)	52 (11.2)
5	Urinary tract infections	19 (4.1)	25 (5.4)	44 (9.5)
6	Intra-abdominal infections	20 (4.3)	13 (2.8)	33 (7.1)
7	Skin and soft tissue infections	23 (4.9)	10 (2.2)	33 (7.1)
8	GI infections and food poisoning	12 (2.6)	20 (4.3)	32 (6.9)
9	Brucellosis	21 (4.5)	8 (1.7)	29 (6.2)
10	Cardiovascular infections	24 (5.2)	1 (0.2)	25 (5.4)
11	Bone and joint infections	13 (2.8)	8 (1.7)	21 (4.5)
12	Sepsis and septic shock	9 (1.9)	10 (2.2)	19 (4.1)
13	Acute exanthematous viral infections	11 (2.4)	1 (0.2)	12 (2.6)
14	Parasitic infections, including echinococcosis	8 (1.7)	3 (0.6)	11 (2.4)
15	Viral hepatitis	8 (1.7)	3 (0.6)	11 (2.4)
16	Fungal infections	9 (1.9)	2 (0.4)	11 (2.4)
17	Viral hemorrhagic fever	6 (1.3)	4 (0.9)	10 (2.2)
18	Leishmaniasis	6 (1.3)	1 (0.2)	7 (1.5)
19	Other categories	22 (4.7)	5 (1.1)	27 (5.8)

*URTI, Upper Respiratory Tract Infection; CNS, Central Nervous System; GI, Gastrointestinal; HIV, Human Immunodeficiency Virus*.

* *Numbers are represented by the number of reported errors with specific characteristics (percent). The denominator that was used for calculating all the percentages was the total number of reported errors included in the study (n = 465)*.

**Table 3:** Frequency of delayed or missed diagnoses, and misdiagnoses in the categories of infectious diseases that were most frequently affected by errors.

	**Delayed or missed diagnosis**	**Misdiagnosis**		**Total cases**
		**Mistaken for another disease**	**Misdiagnosed instead of another disease**	
URTIs and bronchitis	6 (8.7)^*^	17 (24.3)	46 (66.7)	69 (100)
Tuberculosis	43 (64.2)	8 (11.9)	16 (23.9)	67 (100)
Pleuro-pulmonary infections	18 (33.3)	11 (20.4)	25 (46.3)	54 (100)
CNS infections	25 (48.1)	14 (26.9)	13 (25)	52 (100)
Urinary tract infections	12 (27.3)	7 (15.9)	25 (56.8)	44 (100)
Intra-abdominal infections	11 (33.3)	9 (27.3)	13 (39.4)	33 (100)
Skin and soft tissue infections	15 (45.5)	8 (24.2)	10 (30.3)	33 (100)
GI infection and food poisoning	2 (6.3)	10 (31.3)	20 (62.5)	32 (100)
Brucellosis	17 (58.6)	4 (13.8)	8 (27.6)	29 (100)
Cardiovascular infections	17 (68)	7 (28)	1 (4)	25 (100)

*URTI, Upper Respiratory Tract Infection; CNS, Central Nervous System; GI, Gastrointestinal*.

* *Numbers are represented by the number of reported errors with specific characteristics (percent)*.

**Figure 1:**
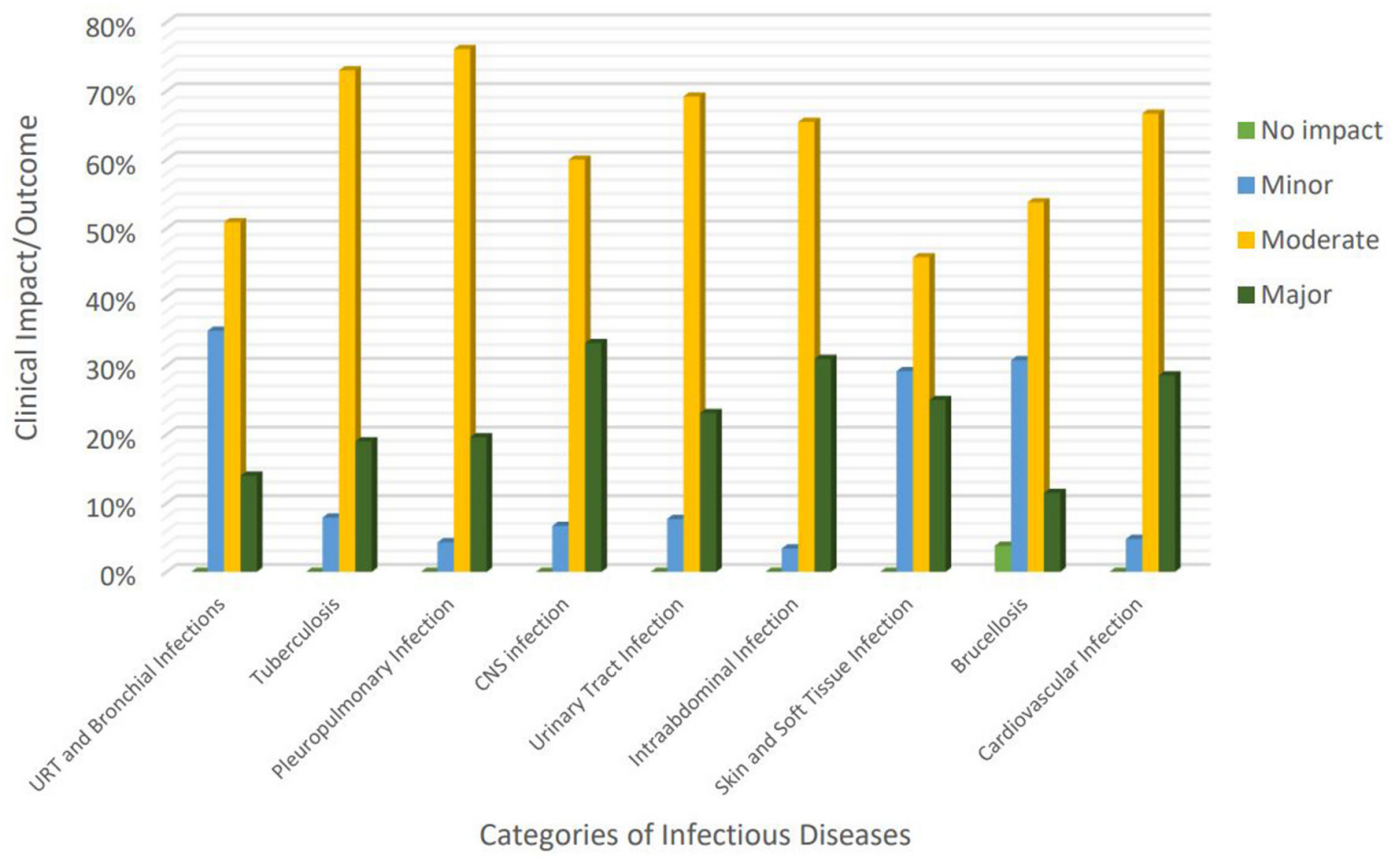
Clinical impact of diagnostic errors regarding the most commonly reported categories of infectious diseases. URTI, Upper Respiratory Tract Infection; CNS, Central Nervous System; GI, Gastrointestinal.

Major impact occurred significantly more frequent in patients who experienced tuberculosis (TB)-related errors (odds ratio [OR]: 2.4, 95% confidence interval [CI]: 0.9–5.7; *P* value: 0.04) and intra-abdominal infection (IAI)-related errors (OR: 7.2, 95% CI: 0.9–53.8; *P* value: 0.02) (Table 4, Figure 1).

**Table 4:** The asociation of errors in each category of infectious diseases with major impact on patients' outcomes (as dependent variable).

**Variables**	**OR**	**95% CI**	***P*** **-value**
URTIs and Bronchitis	0.29	0.16–0.52	<0.001
Tuberculosis	**2.40**	0.99–5.75	0.04
Pleuro-pulmonary Infections	2.76	0.96–7.89	0.05
CNS Infections	2.50	0.87–7.17	0.78
Urinary Tract Infections	3.03	0.91–10.07	0.05
Intra-abdominal Infections	**7.31**	0.98–54.27	0.02
Skin and Soft Tissue Infections	0.51	0.23–1.15	0.10
GI Infection and Food Poisoning	0.27	0.13–0.58	<0.001
Brucellosis	0.44	0.19–1.01	0.04
Cardiovascular Infections	5.12	0.68–38.46	0.78

*OR, odds ratio; CI, confidence interval; URTI, Upper Respiratory Tract Infection; CNS, Central Nervous System; GI, Gastrointestinal*.

**Figure 2:**
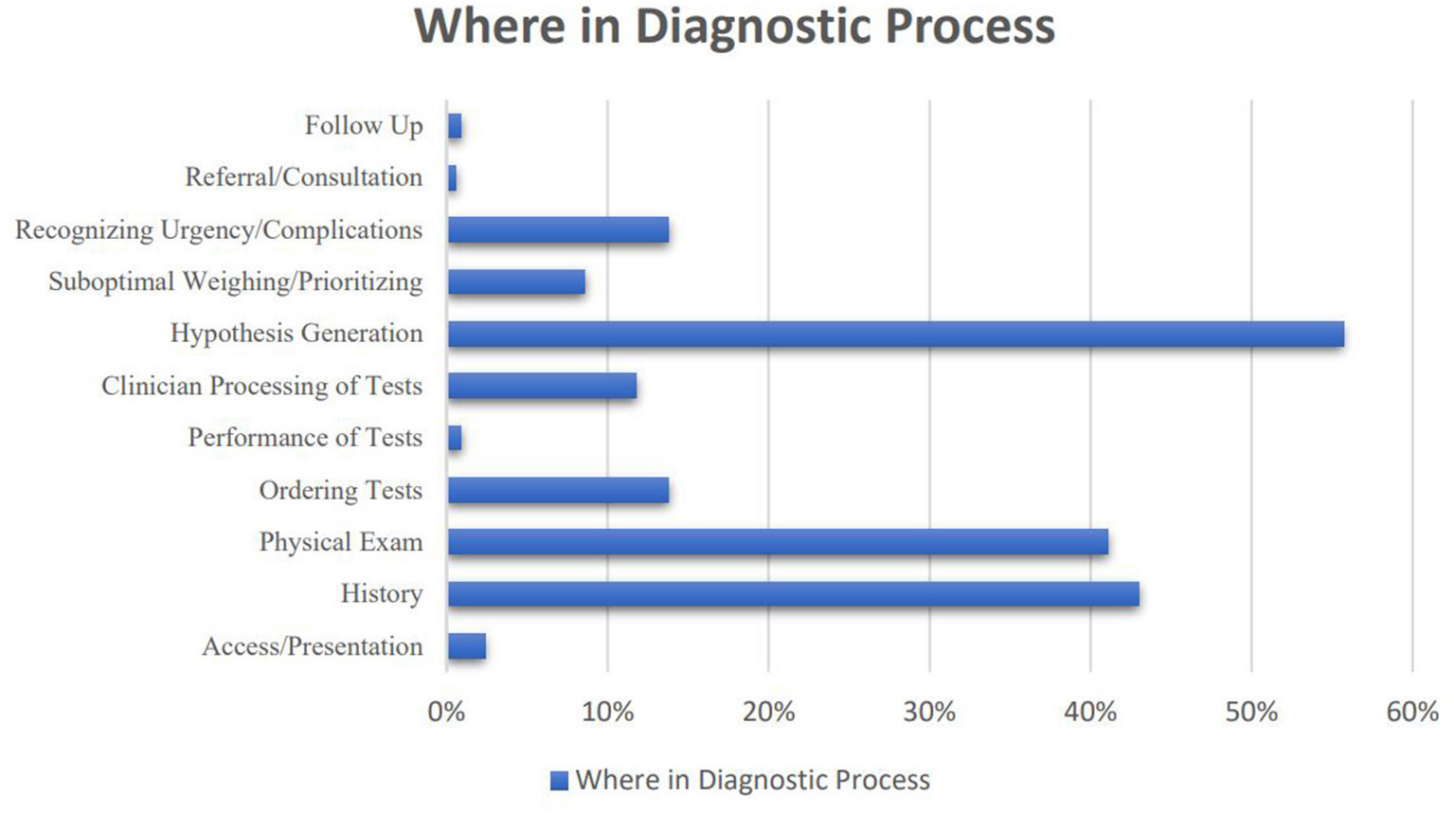
Where errors occurred in the diagnostic process.

Hypothesis generation was the stage at which errors most frequently occurred (*n* = 259, 55.7%), followed by history taking (*n* = 200, 43%), physical examination (*n* = 191, 41.1%), recognizing an urgency or complication (*n* = 65, 14%), and ordering tests (*n* = 65, 14%) (Figure 2).

One hundred thirty-three (65.8%) errors that occurred in hypothesis generation were associated with errors in history taking, 112 (57.7%) physical examination, and 74 (28.6%) both history taking and physical examination.

## Discussion

Analyzing a total of 465 errors in diagnosing infectious diseases, the most common infectious diseases affected by diagnostic errors were upper respiratory tract infections (URTIs), tuberculosis (TB), pleuro-pulmonary infections, central nervous system (CNS) infections, and urinary tract infections (UTIs). TB-related errors and intra-abdominal infection (IAI)-related errors were associated with a significantly higher frequency of major impact on clinical outcome. To the best of our knowledge, this is the largest report of errors in diagnosing infectious diseases to date which also focused on where in the diagnostic process the errors occurred.

As noted in previous reports on medical errors, infectious diseases are among the main categories of illnesses affected by diagnostic errors (17). Pneumonia, TB, diarrhea with dehydration, malaria, CNS infections, infective endocarditis, and abscesses were reported as the categories of infectious diseases which are more frequently prone to harmful diagnostic errors (11, 15, 18–20).

Benign viral infections have been reported as the most common disease categories that were affected by misdiagnosis in primary care (21). Viral infections including viral URTIs are frequently misdiagnosed as bacterial infections that could potentially lead to unnecessary or inappropriate antibiotic prescriptions (17, 22). While most respiratory tract infections are of viral origin and self-limiting, there is clear evidence that antibiotics are heavily overprescribed for respiratory diseases. Inappropriate antibiotic use contributes to the promotion of resistant microorganisms and unnecessarily exposes patients to side effects which both result in unnecessary costs (23). Our study also showed the frequent occurrence of misdiagnosis regarding URTIs. In one-third of URTI-related errors, the wrong causative pathogen was considered. Nevertheless, these errors occurred most often because URTIs were misdiagnosed instead of other infectious or non-infectious diseases. This could be attributed to the frequent use of an intuitive approach of thinking in making such diagnoses. Despite the fact that patient presentation does not always fit into the classic clinical picture of illness, physicians often rely on fast intuition-based thinking that is based on experience and accumulated judgment instead of analytical clinical reasoning (24). Although works well most of the time, the former strategy is much more prone to error (24). For example, jumping to the diagnosis of viral pharyngitis only because it is more common and presents with fever, fatigue and sore throat might cause one to neglect to consider other more serious diseases (e.g., Crimean Congo hemorrhagic fever). Experienced physicians are aware of the limitations of the intuitive approach. Thus, when it is appropriate, they try to “slow down” and devote more time to analyze available clinical and para-clinical data (25).

An important point regarding the impact of errors in diagnosing infectious diseases is that they not only affect the patients but also the community health. This is related to the contagious nature of many infectious diseases that if missed or diagnosed with delay, necessary measures may not be taken to contain the spread of the infectious agents.

TB, as a highly contagious and potentially fatal infectious disease, was among the most common infectious diseases that were missed or diagnosed with delay. Although TB is endemic in Iran and the results of our study may not be representative of all other countries, previous studies also emphasized the challenge of misdiagnosis and delayed diagnosis of TB patients (25, 26). Missed or delayed diagnosis of TB can be catastrophic because it affects patients and the community through delayed treatment, the increased period of infectivity, increased transmission of disease, and increased medical costs and mortality (27). In our study, TB-related errors were more often associated with death or serious impact. We previously showed that failures in the diagnosis of TB occur mainly in the hypothesis generation stage (i.e., delay or failure in considering the diagnosis) (25). Thus, physicians should always consider it on the list of differential diagnoses in the patients who present with compatible clinical syndromes, particularly in the TB-endemic regions.

Pleuro-pulmonary and CNS infections ranked third and fourth among infectious disease categories which were affected by diagnostic errors.

Globally, lower respiratory infections and TB are responsible for about 50 age-adjusted death per 100,000 population and more than 2,000 age-adjusted years of life lost (YLLs) per 100,000 (i.e., the second leading cause of YLLs) (28). It is difficult to estimate whether and how many diagnostic errors are responsible for the poor outcomes related to pleuropulmonary infections. Many serious or self-limiting medical conditions can look like pneumonia upon initial examination. Autopsy-based studies showed that pneumonia is among the potentially treatable diseases that are most frequently under- or over-diagnosed (29). In our survey, some of the conditions that pneumonia was mistaken for included upper respiratory infections, congestive heart failure, pulmonary thromboembolism, lung cancer, and asthma. On the other hand, the diagnosis of pneumonia was misdiagnosed instead of other potentially life-threatening conditions in about half of the reported cases. As a common and serious disease, misdiagnosis of pneumonia can either lead to a dangerous delay in treatment or missing other serious conditions.

CNS infections are among the most serious categories of infectious diseases that are associated with significant morbidity and mortality. Any delay in the diagnosis and treatment of most types of CNS infections can lead to devastating consequences. There are several case reports and series found in the literature describing errors in diagnosing CNS infections (30, 31). We previously highlighted a high rate of errors in the diagnosis of patients with CNS infections and the adverse impact of these errors on patients' clinical outcomes (19). Ordering appropriate tests/imaging was the most common failure in the diagnostic process of patients with community-acquired CNS infections (19). Diagnosis of CNS infections was misdiagnosed instead of other diseases in 25% of reported cases in our study; however, missed or delayed diagnoses, or being mistaken for other diseases were reported more frequently. When facing a patient who presents with a clinical syndrome suspected of a potentially life-threatening 0 such as CNS infections, erring on the side of caution and considering the most critical illnesses might be more acceptable than missing a benign or self-limiting condition; however, the patient who does not match the presumed diagnoses deserves diagnostic reassessment (32).

In almost all the studies that evaluated clinical vs. autopsy diagnoses, a significant incidence of discrepancies has been found. Not all the errors or discrepancies carry equal weight: some are relatively inconsequential, but others have considerable impact and might have influenced patient survival if recognized during life (18). Many diagnostic errors identified in our study were associated with a moderate to serious impact on patients' outcomes. Since the human memory tends to better remember highly salient events (33), the self-report data of our study might be affected by the memory bias of the participants. Nevertheless, it could also have an important message that errors in diagnosing infectious diseases are an important source of serious and potentially preventable misdiagnosis-related harm to the patients and community, and result in death or disability in a significant proportion of patients.

Diagnostic errors are often under-recognized because they are difficult to measure and keep track of. A gap frequently exists between the time they occur and when they are detected (34). We identified that the highest rate of errors in diagnosing infectious diseases occurs in the hypothesis generation, followed in decreasing order by history taking, physical examination, recognizing an urgency or complications, and ordering tests. Of 583 reported cases of diagnostic errors in internal medicine in another physician survey, most errors occurred in the testing phase (failure to order, report, and follow-up laboratory tests) and clinical assessment (failure to consider and overweighing competing diagnoses) (15). The difference might be related to the most common categories of illnesses which were misdiagnosed that were pulmonary thromboembolism, drug reactions or overdose, lung and colorectal cancer, and acute coronary syndrome in their study while URTIs, TB, pleuropulmonary infections, CNS infections, and UTIs in our survey. Shortcomings in generating a diagnostic hypothesis could be the result of faulty thinking in clinical assessment or insufficient knowledge. However, it is often a misuse of knowledge and not a lack of knowledge that leads to diagnostic errors (32). It could also be related to incomplete data acquisition from the patients. We found 66 and 58% of errors in the hypothesis generation were associated with failure to obtain clinical information through history taking and physical examination, respectively. Accordingly, improving the art of diagnosis in infectious diseases needs to focus on both reasoning and data acquisition.

Our study had several limitations. The results of our study might be affected by hindsight bias. For assessing the quality of a decision, one must use the information available at the time, filtering out everything he knows about the outcome. The estimated likelihood and severity of diseases influence the measurement of diagnostic error (16). Unless a physician keeps a journal or diary about errors he made or witnessed, this is an inevitable psychological phenomenon to view events as more predictable than they really are. Memory bias could also affect the results of our study. To reduce this bias, we presented our participants with a one-page comprehensive list of infections and infectious diseases to help them remember errors regarding different categories of infectious diseases. However, this bias frequently occurs in self-report surveys. We also found substantial variability in the details of case descriptions provided by respondents. Furthermore, some of the cases described by respondents could not be considered errors and were including different illnesses on the list of differential diagnoses in the early assessment of patients. Thus, we excluded them from the analysis.

## Conclusion

Our study shows that among infectious diseases, upper respiratory tract infections (URTIs), tuberculosis (TB), pleuro-pulmonary infections, central nervous system (CNS) infections, and urinary tract infections (UTIs) are more frequently prone to diagnostic errors. While errors occurred at any stage in the diagnostic process, in the majority of cases, the failure occurred in the hypothesis generation, history taking, and physical examination. Failure in generating diagnostic hypotheses was frequently preceded by incomplete history taking and physical examinations. Hence, we conclude that improving the art of diagnosis in infectious diseases needs to focus on both reasoning and data acquisition.

Errors in diagnosing infectious diseases not only affect the patient but also the community health. While a substantial proportion of errors in diagnosing infectious diseases were associated with significant impacts on patients' outcomes, errors related to TB and intra-abdominal infections more often resulted in poor clinical outcomes. Thus, contagious and potentially life-threatening infectious diseases should always be kept in mind when listing differential diagnoses for patients with compatible clinical syndromes.

Errors in diagnosing infectious diseases included both missed or delayed diagnosis of infectious diseases as well as misdiagnosis of an infectious disease instead of a non-infectious disease. Although erring on the side of caution and considering the most critical illnesses such as CNS infections might be more acceptable than missing a benign or self-limiting condition, patients who do not match the presumed diagnoses deserve diagnostic reassessment.

## Data Availability Statement

The raw data supporting the conclusions of this article will be made available by the authors, without undue reservation.

## Author Contributions

MH, AS, and MS performed the research and analyzed the data. FS, HRN, MNN, and MSS designed the study. MH wrote the first draft of the paper. FS, HRN, and MSS revised the paper. FS, MH, HRN, MNN, MSS, AS, and MS have read and approved the final manuscript.

## Conflict of Interest

The authors declare that the research was conducted in the absence of any commercial or financial relationships that could be construed as a potential conflict of interest.

## Publisher's Note

All claims expressed in this article are solely those of the authors and do not necessarily represent those of their affiliated organizations, or those of the publisher, the editors and the reviewers. Any product that may be evaluated in this article, or claim that may be made by its manufacturer, is not guaranteed or endorsed by the publisher.
